# Quality of life outcomes in patients undergoing knee replacement surgery: longitudinal findings from the QPro-Gin study

**DOI:** 10.1186/s12891-020-03456-2

**Published:** 2020-07-04

**Authors:** Paola Siviero, Anna Marseglia, Carlo Biz, Augusto Rovini, Pietro Ruggieri, Roberto Nardacchione, Stefania Maggi

**Affiliations:** 1 National Research Council, Neuroscience Institute - Aging Branch, via Giustiniani, 2, 35128 Padova, Italy; 2 Aging Research Center, Karolinska Institutet and Stockholm University, SE-171 77 Stockholm, Sweden; 3 Orthopaedic Clinic, Department of Surgery, Oncology and Gastroenterology DiSCOG, University of Padua, via Giustiniani 2, 35128 Padova, Italy; 4 Abano General Hospital, Abano Terme, Piazza Cristoforo Colombo 1, 35031 Abano Terme, PD Italy

**Keywords:** Quality of life, Osteoarthritis, Knee replacement, Arthroplasty, Longitudinal study

## Abstract

**Background:**

Many patients report postoperative pain, limited improvement in physical function and poor quality of life (QOL) after knee replacement surgery. Our study uses baseline predictors of change to investigate the QOL of patients with knee osteoarthritis 3-months after knee replacement surgery.

**Methods:**

A prospective observational study was designed to evaluate patients (*n* = 132) scheduled for uni-compartmental or total knee replacement surgery who were assessed at baseline (preoperatively) and 3-months after. Physical and mental endpoints based on the component scores of the SF-12 and on the Western Ontario and McMaster Universities Arthritis (WOMAC) index were used to investigate patients’ QOL. Generalised estimating equation methodology was used to assess patients’ baseline characteristics (age, sex, education, body mass index (BMI), comorbidity, depressive symptoms, cognitive impairment, smoking/alcohol and type of surgery), the study endpoints and their changes over a 3-month post-surgery period. Stratified analyses by rehabilitation status after discharge were performed.

**Results:**

Longitudinal data analysis showed that the baseline factors associated with improvement in general QOL at the 3-month post-surgery assessment were higher BMI, a high comorbidity, total (as opposed to unicompartmental) knee replacement and low education level. Data analysis of the patients who underwent rehabilitation after discharge revealed that the current smokers’ physical QOL worsened over time. The general QOL was unchanged over time in the presence of depressive symptomatology.

**Conclusions:**

These findings underline the importance of using comprehensive assessment methods to identify factors affecting functionality and QOL, and developing interventions to improve the health/wellbeing of patients after knee replacement.

## Background

The primary pathology requiring knee replacement surgery is knee osteoarthritis (OA). Rising life expectancies and the global obesity epidemic are leading to a rapid increase in the prevalence of knee OA and, therefore, to a greater demand for knee replacement surgery. These patterns are causing and will continue to cause future important implications for health care expenditures [1]. Acute and persistent pain (usually at night), severe functional disability and the failure of non-surgical treatments are the deciding factors for surgical intervention [2]. Two principal surgical options for late-stage medial compartment OA of the knee are total knee arthroplasty (TKA) or unicompartmental knee arthroplasty (UKA), in which only the damaged compartment of the knee is replaced [3, 4]. Surgeons often disagree about the best choice of surgery for these patients, having often identical pathologies, which has caused variations in implants and treatment [5].

Assessments of knee replacement surgery outcomes traditionally use revision of knee joint replacement as an endpoint; however, other physical variables may also be assessed [1]. Although significant advancements have been made in both surgical techniques and prosthesis placement procedures, many patients continue to report postoperative pain, limited improvement in physical function, modest clinical benefit of UKA over TKA and poor quality of life (QOL) [6]. In several studies furthermore, it has been shown that younger age, in which UKA is more indicated, is associated with higher revision rates [7–9]. On the other hand, octogenarians and nonagenarians undergoing elective TKA experience relatively high rates of complications, even if most of these are minor. At the same time, ADL-dependent patients and those with a history of congestive heart failure or chronic obstructive pulmonary disease are more likely to experience unplanned readmission [10].

These suboptimal results may not be entirely due to the surgical procedure itself, to surgery-related complications, or to physical comorbidities. The patient’s pre-operative level of pain and psychological profile, or other variables may be involved [11].

The multi-dimensional QOL is a measure that places heavy emphasis on “health”, which is not characterised as the complete absence of disease but as a condition of general physical, social and mental well-being [12]. When that type of approach is used, the success of a knee replacement procedure and the QOL of a patient after surgery must be evaluated not only on the basis of physical function but also on psychological and social factors that could influence the surgery’s outcomes. To our knowledge and despite data underlining the importance of using a multi-dimensional approach, there have been few studies of the outcomes of different types of knee replacement, and findings gathered until now tend to be conflicting [1, 11, 13].

The primary aim of the present analysis was to use physical and mental endpoints to investigate patient QOL at the time of knee replacement surgery and 3-months later. The secondary aim was to identify the baseline predictors of change in QOL in the patients who were stratified according to their participation or non-participation in a rehabilitation program after discharge.

## Methods

### Study design and sample characteristics

The competent Ethical Committee of Padova approved the Quality of Life in Knee Prostheses (QPro-Gin) project (identifier: 258 OS), which is a single-centre prospective study. The inclusion criteria for this study were the following: age ≥ 40 years; OA or osteonecrosis (one compartment or both for UKA and TKA, respectively); and being scheduled for unicompartmental or total knee replacement surgery at the Orthopaedics and Traumatology Unit of the Abano Terme (Italy) General Hospital. The exclusion criteria included: diagnosis of inflammatory arthritis, haemochromatosis, chondrocalcinosis or haemophilia; multi-compartment disease; unsuccessful correctional osteotomy or ipsilateral UKA; symptomatic knee instability or anterior cruciate ligament deficiency; immobility or other neurological conditions affecting musculoskeletal function.

All subjects participating in this study signed a consent form, which was obtained on the day the baseline data were collected before undergoing surgery. The type of knee replacement surgery was categorised as UKA (the MAKO Stryker, assisted surgery, Fort Lauderdale, USA and the Oxford mobile-bearing knee implant, Zimmer Biomet Ltd. Oxford) versus TKA (the Vanguard prosthesis, Zimmer Biomet, Warsaw, USA).

The indications for surgery were debilitating knee pain, defined as severe persistent pain, causing important reduction of knee functionality during basic activities of daily living (ADLs), in combination with isolated medial unicompartmental OA with grade 3 loss of articular cartilage according to the Kellgren and Lawrence grading scale [14] or spontaneous medial osteonecrosis of the femur with grade 3 loss of articular cartilage or minor subchondral collapse for UKA and primary end-stage diffused symptomatic OA of the knee for TKA, respectively. Standard radiographic evaluation was carried out preoperatively on weight-bearing radiographs: anteroposterior, Rosenberg, lateral and skyline views.

### Surgical techniques, post-operative treatment and rehabilitation program

All operative procedures, performed with a tourniquet, were carried out by one of two senior authors, both with over 10 years of experience performing both UKA and TKA. Plexus anaesthesia was performed entailing a regional block, which involved both sciatic and femoral nerves. Sedation was used when necessary. Intravenous Cefazolin was used as perioperative prophylaxis (1 g 4 times/day) and continued for a 24 h period after surgery. Postoperative antithrombotic therapy (Natrium Enoxaparin) was given until full free weight bearing was achieved.

The same standardized post-operative physical rehabilitation protocol, which is still applied routinely by physiotherapist teams, was used for each patient regardless of the type of implants, described as follows. Structured physical therapy was begun the day after surgery and continued during the in-hospital stay for a week in case of UKA, and 2 weeks for TKA. The patients having undergone UKA were instructed to sit up at bedside the evening of their surgery and to begin ambulating with assistance the day after surgery. For patients having undergone TKA, active range of motion (ROM) was encouraged and full weight-bearing ambulation was allowed on post-operative day 2 when quadricep inhibition from the femoral nerve block had ceased. Each physiotherapy session lasted 25 min every day, and all rehabilitation was performed by the same multidisciplinary hospital team. Patients were discharged to their own homes after adequate mobilisation with the use of crutches, and independent ascent and descent of stairs. They were encouraged to do specific knee ROM exercises and to seek formal physical therapy on an outpatient basis two or three times per week for the first 3 months. No patients were discharged to a rehabilitation centre or other skilled nursing facilities.

### Patient assessment

There were two time points for data collection: at baseline (during the scheduled hospitalisations prior to the knee-replacement surgery) and 3-months after surgery. Post-surgery, each participant underwent a standard clinical rehabilitation program in place at Abano Terme General Hospital, as described above. According to the inclusion and exclusion criteria, the eligible participants were selected from the surgery list; each patient included in this study was assessed by a board-certified neuropsychologist. The baseline evaluations were carried out through January and May 2013, and the 3-month post-surgery evaluations were carried out from April 2013 to July 2013.

### Data collection

Information on the participants’ demographic variables, medical history and medication, lifestyle (smoking and alcohol use) and type of surgery scheduled (UKA or TKA) was collected at baseline. Data on pain, stiffness and disability due to knee OA were collected at baseline and 3 months after surgery. Information on participation in a rehabilitation program after discharge was collected at the 3-month post-surgery assessment.

### Measures

#### Endpoints

The QPro-Gin study’s primary endpoint was health-related QOL as measured by the Short-Form General Health Survey (SF-12) Questionnaire. The patients were asked to fill out the Physical (PCS) and Mental (MCS) components, which are considered QOL indicators [15].

The QPro-Gin study’s secondary endpoint was the self-reported assessment of pain, stiffness and disability in the knee as measured using the Western Ontario and McMaster Universities Arthritis Index (WOMAC) [16], an instrument that evaluates three dimensions (pain, stiffness and physical function).

#### Information about the sample population

Information was gathered on patient age (categorised as < 65 versus ≥65), sex, and educational level (defined as low education = elementary or middle school versus high education = high school or university degree). The patients’ Body Mass Index (BMI) was categorised as BMI < 30 kg/m^2^ versus BMI ≥30, the latter defined obesity [17]. Scores of 6 or above on the Short Form Geriatric Depression Scale (GDS) indicated the presence of depressive symptoms [18]. Cognitive impairment was defined as Mini-Mental State Examination (MMSE) score < 24 [19].

Comorbidity was defined as the presence of ≥4 (median value) coexisting disorders of the sensory, respiratory, cardiovascular, gastrointestinal, endocrine/metabolic, neurological, urogenital/gynaecological, immunologic-rheumatic, musculoskeletal, psychiatric systems, or cancer. Medication used (dichotomised as “yes” or “no”) referred to corticosteroid/anti-inflammatory/analgesic drugs and to medicines for cardiovascular, gastrointestinal, metabolic, neurological, bone and other diseases. The lifestyle behaviours that were assessed were smoking status (current versus not current smoking) and alcohol consumption (current user versus non-user).

At the 3-months post-surgery assessment, patients were asked if they had followed the rehabilitation program after discharge (dichotomised as “yes” or “no”).

### Statistical analysis

#### Calculating the sample size

The sample size needed for our analysis was calculated a priori in accordance with Poitras et al.’s study [20]. Because 3 months after joint replacement, the mean PCS of the SF-12 of 61 patients was 38.7 ± 9.5, we calculated that a sample size of 120 was required to ensure a probability of 0.90 to produce a two-sided 95% confidence interval with a distance from the mean to the limit that was less than or equal to 2. A 20% overestimation, 151 patients, were thus enrolled to allow for the possibility of dropouts.

#### Baseline sample characteristics

Continuous variables were expressed as mean and standard deviation (SD) or as median and interquartile range (IQR) if non-normally distributed; categorical variables were reported as percentages. Comparisons of normally distributed characteristics were carried out using the generalised linear model procedure after homoscedasticity was verified using Levine’s test (in case of heteroscedasticity, Welch’s analysis of variance was applied).

#### Change over time

The change in the endpoints over the 3-month after surgery period was assessed using the paired t-test.

Generalised estimating equation (GEE) models for longitudinal data analysis were used to assess the influence of baseline *characteristics* (age, sex, education level, BMI, depressive symptoms, cognitive impairment, number of comorbid diseases, smoking and alcohol statuses, type of surgery) on change over the 3-month post-surgery period [21].

GEE models estimated β-coefficients, 95% confidence intervals (95% CI), and standard error (SE) of the cross-sectional (between-individuals) and longitudinal (within-individual) associations between independent variables and endpoints. Each GEE model also included an interaction term between time and the independent variables.

Predicting models of endpoints stratified by rehabilitation after discharge were developed.

*P* values of 0.05 for two-tailed tests were considered significant. Statistical analyses were performed using SAS software (SAS Institute Inc., Cary, NC, USA) version 9.4.

## Results

One hundred fifty-one patients (41 men = 27.2%; 110 women = 72.8% with a mean age of 68.3 years (SD 8.3) scheduled for knee replacement surgery were considered eligible to participate in the study. Nineteen (12.6%) withdrew from the study after the baseline assessment: 6 freely chose to abandon the study, 12 were lost, and 1 underwent a different type of surgery. Their characteristics were nevertheless similar to those of the 132 (87.4%) who did complete the two assessments upon which the analyses were based. Among them, 47 were treated by UKA (24 MAKO) while 85 by TKA.

### Patients’ characteristics at baseline

The baseline characteristics of the participants are presented in Table 1. Most of the patients were female and overweight who underwent a TKA procedure; they had on average ≥ 4 comorbid conditions, in particular musculoskeletal and cardiovascular diseases.

**Table 1:** Patients’ characteristics at baseline

	(***n*** = 132)
**Age,** mean ± SD, years	67.9 ± 8.6
**Female Sex**, n (%)	97 (73.5)
**Low education,** n (%)	86 (65.2)
**BMI,** mean ± SD, kg/m^2^	28.7 ± 4.4
**GDS,** median (IQR)	1 (0–5)
**MMSE,** median (IQR)	29 (28–30)
**Diseases,** n (%)	
**Sensory**	53 (40.2)
**Respiratory**	25 (18.9)
**Cardiovascular**	97 (73.5)
**Gastrointestinal**	57 (43.2)
**Endocrine**	30 (22.7)
**Metabolic**	50 (37.9)
**Neurological**	26 (19.7)
**Urogenital/gynaecological**	22 (16.7)
**Immunologic-rheumatic**	8 (6.1)
**Musculoskeletal**	124 (93.9)
**Psychiatric**	39 (29.5)
**Oncological**	10 (7.6)
**Other**	10 (7.6)
**Comorbidity (No. of diseases),** median (IQR)	4 (3,5)
**Baseline Medications**, n (%)	122 (92.4)
**Corticosteroid/anti-inflammatory/analgesic**	58 (43.9)
**for cardiovascular diseases**	84 (63.6)
**for gastrointestinal diseases**	42 (31.8)
**for metabolic diseases**	58 (43.9)
**for neurological diseases**	43 (32.6)
**for bone diseases**	24 (18.2)
**for other diseases**	20 (15.2)
**Current smoker,** n (%)	17 (12.9)
**Current alcohol user,** n (%)	105 (79.5)
**Surgery type**, n (%)	
UKA	47 (35.6)
TKA	85 (64.4)

Abbreviation: *SD* standard deviation, *IQR* inter quartile range, *BMI* Body Mass Index, *GDS* Geriatric Depression Scale, *MMSE* Mini-Mental State Examination, *UKA* unicompartmental knee arthroplasty, *TKA* total knee arthroplasty

GDS ranges from 0 [best] to 15 [worst]

MMSE ranges from 0 [worst] to 30 [best]

Table 2 presents the baseline distribution of SF-12 and WOMAC-index according to patients’ characteristics.

**Table 2:** Baseline distribution of sf-12 and womac-index according to patients’ characteristics

	(***n*** = 132)	SF 12-PCS		SF 12-MCS		WOMAC-Index	
	N (%)	Mean ± SD	P	Mean ± SD	P	Mean ± SD	P
**Age**			0.318		0.900		0.925
< 65 years	42 (31.8)	34.4 ± 9.3		48.2 ± 12.4		45.9 ± 14.9	
≥ 65 years	90 (68.2)	36.1 ± 8.8		48.0 ± 10.5		45.6 ± 16.6	
**Sex**			**0.040**		**0.002**		**< 0.001**
Male	35 (26.5)	38.2 ± 9.4		53.0 ± 10.8		37.4 ± 14.3	
Female	97 (73.5)	34.6 ± 8.7		46.3 ± 10.7		48.7 ± 15.6	
**Education**			0.159		**0.001**		**0.009**
Low	86 (65.2)	34.8 ± 8.8		45.8 ± 11.3		48.4 ± 16.0	
High	46 (34.8)	37.1 ± 9.3		52.3 ± 9.4		40.7 ± 14.9	
**BMI**			**0.001**		0.635		0.074
< 30 kg/m^2^	87 (65.9)	37.4 ± 8.5		48.4 ± 11.1		43.9 ± 16.0	
≥ 30 kg/m^2^	45 (34.1)	31.9 ± 8.8		47.4 ± 11.3		49.2 ± 15.6	
**Depressive symptoms (GDS ≥ 6)**			**0.005**		**< 0.001**		**< 0.001**
No	102 (77.3)	36.6 ± 9.2		51.1 ± 9.3		42.7 ± 14.9	
Yes	30 (22.7)	31.9 ± 7.3		37.7 ± 10.7		55.8 ± 15.7	
**Cognitive impairment (MMSE < 24)**			0.883		0.663		0.529
No	121 (91.7)	35.5 ± 9.2		48.2 ± 10.8		45.4 ± 16.1	
Yes	11 (8.3)	35.9 ± 5.9		46.6 ± 14.7		48.6 ± 14.8	
**Comorbidity**			0.191		**0.033**		0.699
< 4 diseases	44 (33.3)	37.0 ± 8.9		51.0 ± 10.4		46.5 ± 17.9	
≥ 4 diseases	88 (66.7)	34.0.8 ± 9		46.6 ± 11.2		45.3 ± 15.1	
**Current smoker**			0.387		0.828		0.400
No	115 (87.1)	35.3 ± 9.1		48.1 ± 10.7		46.2 ± 15.9	
Yes	17 (12.9)	37.3 ± 7.7		47.5 ± 13.7		42.6 ± 16.6	
**Current alcohol user**			0.097		0.101		**0.005**
No	27 (20.5)	33.0 ± 8.0		44.9 ± 10.6		52.1 ± 11.5	
Yes	105 (79.5)	36.2 ± 9.1		48.9 ± 11.1		44.1 ± 16.6	
**Surgery type**			0.574		0.159		**0.016**
UKA	47 (35.6)	36.2 ± 8.2		46.2 ± 11.0		41.2 ± 13.8	
TKA	85 (64.4)	35.2 ± 9.4		49.1 ± 11.1		48.2 ± 16.7	

Abbreviation: *SD* standard deviation, *SF-12* Short-Form General Health Survey, *PCS* physical component scores, *MCS* mental component scores, *WOMAC* Western Ontario and McMaster Universities Arthritis Index, *BMI* Body Mass Index, *GDS* Geriatric Depression Scale, *MMSE* Mini-Mental State Examination, *UKA* unicompartmental knee arthroplasty, *TKA* total knee arthroplasty

SF-12 (PCS, MCS) ranges from 0 [worst] to 100 [best]

WOMAC-Index is normalised to 100, ranges from 0 [best] to 100 [worst]

*SF-12* scores showed that the women had worse scores on both components. The patients with low education tended to have worse *MCS* scores, and the obese patients tended to have worse PCS scores with respect to those with a BMI < 30 kg/m^2^. The patients with ≥4 diseases had worse MCS scores. There were no statistically significant differences in the SF-12 scores for age, smoking/alcohol status and type of surgery.

Analysis of the *WOMAC-Index* scores (mean ± SD 45.7 ± 16.0) showed that the women had significantly worse scores. The patients with low education, depressive symptoms who were not currently alcohol users and undergoing TKA surgery tended to have worse scores**.** There were no statistically significant differences in the WOMAC-Index scores for age, BMI, cognitive impairment, comorbidity and smoking habits.

### Change in endpoints

The mean change (Follow-up value – Baseline value) for the PCS was 4.2 (SD 11.3); it was 2.0 (SD 11.3) for the MCS; it was − 23.2 (SD 17.6) for the WOMAC–Index (Table 3). A statistically significant improvement in all end points over time was detected in the population as a whole.

**Table 3:** Change in endpoints

		Baseline	Follow-up: 3 Months	Change: Follow-up - Baseline	***P***
**SF 12-PCS,** n, mean ± SD	132	35.6 ± 9	39.7 ± 8.7	4.2 ± 11.3	**< 0.001**
**SF 12-MCS**, n, mean ± SD**,**	132	48.1 ± 11.1	50 ± 10.7	2.0 ± 11.3	**0.044**
**WOMAC–Index,** n, mean ± SD	132	45.7 ± 16	22.5 ± 14.5	−23.2 ± 17.6	**< 0.001**

Abbreviation: *SD* standard deviation, *CI* confidence intervals, *SF 12* Short-Form General Health Survey, *PCS* physical component scores, *MCS* mental component scores, *WOMAC* Western Ontario and McMaster Universities Arthritis Index, *P p*-value for paired t-test

SF 12 ranges from 0 to 100, with 0 indicating worst quality of life

WOMAC-Index ranges from 0 to 100, with 100 indicating more limitation (pain, stiffness and physical function)

### Association of the baseline *characteristics* with the endpoints

Tables 4, 5 and 6 show the results of the GEE analyses that assessed the influence of the baseline *characteristics* on each endpoint, stratifying the patients according to rehabilitation after discharge. After discharge, 3 months after surgery, 40 of the patients (30% of the study completers) had continued the same rehabilitation program at home.

**Table 4:** SF 12-PCS and associated patients’ characteristics and clinical conditions: multivariable generalised estimating equation models

SF 12-PCS	Total (n = 132)			Rehabilitation program after discharge					
				Yes (***n*** = 40)			No (***n*** = 92)		
	β	95%CI	P	β	95%CI	P	β	95%CI	P
Intercept	40.41	34.77,46.04	< 0.001	43.01	31.41,54.61	< 0.001	39.29	32.84,45.74	< 0.001
Time (3-months post-surgery)	7.09	−0.26,14.45	0.059	0.36	−16.46,17.18	0.967	9.99	1.43,18.55	**0.022**
**Age ≥ 65 year**	3.02	−0.63,6.66	0.105	5.88	−0.31,12.08	0.063	1.60	−2.71,5.92	0.467
Time*Age ≥ 65 year	−5.21	−9.69,-0.74	**0.022**	−12.18	−19.79,-4.57	**0.002**	−1.92	−7.05,3.21	0.463
**Female sex**	−2.96	−6.38,0.46	0.090	−4.83	−11.65,2.00	0.166	−2.13	−6.29,2.02	0.314
Time*Female sex	−1.24	−5.72,3.23	0.586	0.18	−10.49,10.86	0.973	−1.87	−6.92,3.17	0.467
**Low education**	−0.51	−4.23,3.20	0.786	−1.58	−7.01,3.85	0.568	−0.31	−4.75,4.14	0.892
Time*Low education	−0.93	−5.64,3.78	0.698	1.44	−5.89,8.77	0.701	−2.91	−7.93,2.11	0.256
**BMI ≥ 30** kg/m^2^	−4.96	−8.27,-1.65	**0.003**	−6.03	−13.14,1.09	0.097	−5.85	−9.61,-2.10	**0.002**
Time*BMI ≥30 kg/m^2^	4.85	0.96,8.73	**0.015**	7.66	−2.72,18.03	0.148	5.52	1.10,9.94	**0.014**
**Depressive symptoms**	−4.34	−7.60,-1.08	**0.009**	−6.37	−12.41,-0.32	**0.039**	−3.34	−7.24,0.56	0.093
Time*Depressive symptoms	−1.85	−6.03,2.33	0.386	1.57	−6.24,9.38	0.694	−3.00	−7.48,1.48	0.190
**Cognitive impairment**	1.89	−2.01,5.78	0.343	−2.01	−10.74,6.71	0.651	1.94	−2.84,6.73	0.426
Time*Cognitive impairment	3.30	−2.16,8.76	0.236	13.03	−6.37,32.43	0.188	2.65	−2.79,8.09	0.339
**Comorbidity ≥ 4 diseases**	− 2.13	−5.08,0.82	0.157	−3.42	−9.63,2.80	0.281	−1.97	−5.53,1.59	0.278
Time*Comorbidity ≥4 diseases	4.86	0.64,9.09	**0.024**	11.24	3.45,19.02	**0.005**	3.39	−1.40,8.17	0.166
**Current smoker**	2.12	−2.00,6.24	0.313	11.01	1.56,20.46	**0.022**	−0.47	−4.73,3.79	0.829
Time*Current smoker	−3.01	−7.46,1.43	0.184	−16.04	−24.00,-8.08	**< 0.001**	−0.53	−4.88,3.82	0.810
**Current alcohol user**	1.14	−2.29,4.57	0.514	1.59	−4.74,7.92	0.622	2.28	−1.79,6.36	0.273
Time*Current alcohol user	−4.34	−9.09,0.41	0.073	−3.39	−12.40,5.63	0.462	−5.83	−11.09,-0.56	**0.030**
**TKA surgery**	−2.53	−5.58,0.52	0.104	−6.03	−11.65,-0.41	**0.036**	−1.13	−4.66,2.39	0.529
Time*TKA surgery	1.93	−1.62,5.49	0.287	4.55	−1.64,10.75	0.150	1.06	−3.00,5.11	0.609

Abbreviation: β, coefficient regression, *CI* confidence intervals, *SF-12* Short-Form General Health Survey, *PCS* physical component scores, *BMI* Body Mass Index, *TKA* total knee arthroplasty (reference UKA, unicompartmental knee arthroplasty)

SF 12-PCS ranges from 0 [worst] to 100 [best]

**Table 5:** SF 12-MCS and associated patients’ characteristics and clinical conditions: multivariable generalised estimating equation models

SF 12-MCS	Total (n = 132)			Rehabilitation program after discharge					
				Yes (***n*** = 40)			No (***n*** = 92)		
	β	95%CI	P	β	95%CI	P	β	95%CI	P
Intercept	55.94	50.27,61.61	< 0.001	53.37	38.98,67.76	< 0.001	55.51	50.31,60.72	< 0.001
Time (3-months post-surgery)	2.31	−4.84,9.46	0.527	−7.95	−20.26,4.35	0.205	4.34	−4.19,12.87	0.319
**Age ≥ 65** year**s**	3.61	−0.63,7.85	0.095	4.95	−1.94,11.85	0.159	3.64	−1.44,8.71	0.160
Time*Age ≥ 65 years	−2.83	−7.73,2.07	0.258	−2.39	−9.31,4.52	0.498	−2.82	−9.07,3.43	0.376
**Female sex**	−3.10	−6.67,0.47	0.089	1.87	−5.27,9.00	0.608	−4.88	−9.13,-0.63	**0.024**
Time*Female sex	−3.39	−7.44,0.65	0.100	−3.93	−11.33,3.48	0.299	−2.16	−7.28,2.96	0.409
**Low education**	−5.35	−8.52,-2.18	**0.001**	−5.49	−10.60,-0.38	**0.035**	−6.59	−10.81,-2.36	**0.002**
Time*Low education	4.32	0.36,8.28	**0.033**	5.00	−0.49,10.48	0.074	5.80	0.31,11.29	**0.038**
**BMI ≥ 30** kg/m^2^	0.93	−2.41,4.27	0.586	− 2.22	−7.03,2.58	0.364	1.90	−2.25,6.04	0.369
Time*BMI ≥30 kg/m^2^	1.16	−3.11,5.43	0.596	4.59	−2.08,11.25	0.177	0.97	−4.60,6.53	0.734
**Depressive symptoms**	−12.41	−16.51,-8.31	**< 0.001**	−14.51	−20.71,-8.31	**< 0.001**	− 10.23	− 15.99,-4.47	**0.001**
Time*Depressive symptoms	3.22	−1.91,8.36	0.219	−0.13	−7.62,7.37	0.974	3.16	−3.80,10.11	0.374
**Cognitive impairment**	3.86	−2.33,10.05	0.222	11.61	4.03,19.20	**0.003**	0.73	−6.76,8.22	0.849
Time*Cognitive impairment	0.03	−5.73,5.80	0.992	−4.64	−13.82,4.54	0.322	1.18	−6.16,8.51	0.753
**Comorbidity ≥ 4 diseases**	−3.45	−6.83,-0.07	**0.045**	−7.02	−13.43,-0.61	**0.032**	−0.89	−5.26,3.48	0.690
Time*Comorbidity ≥4 diseases	0.35	−3.43,4.13	0.856	6.78	0.23,13.32	**0.042**	−2.48	−7.58,2.62	0.340
**Current smoker**	−1.02	−5.84,3.80	0.678	3.13	−2.19,8.46	0.249	−1.46	−7.99,5.07	0.662
Time*Current smoker	1.52	−4.86,7.91	0.640	−4.70	−12.15,2.75	0.217	3.82	−4.15,11.79	0.347
**Current alcohol user**	−0.68	−4.55,3.19	0.731	0.30	−7.68,8.28	0.941	−0.36	−4.70,3.98	0.871
Time*Current alcohol user	2.10	−2.69,6.89	0.390	9.09	4.47,13.70	**< 0.001**	−0.08	−5.85,5.68	0.978
**TKA surgery**	0.88	−2.54,4.30	0.614	0.84	−5.12,6.79	0.783	1.58	−2.45,5.62	0.442
Time*TKA surgery	−3.01	−6.93,0.90	0.132	−2.70	−8.72,3.32	0.380	− 3.95	−9.03,1.13	0.128

Abbreviation: β, coefficient regression, *CI* confidence intervals, *SF-12* Short-Form General Health Survey, *MCS* mental component scores, *BMI* Body Mass Index, *TKA* total knee arthroplasty (reference UKA, unicompartmental knee arthroplasty)

SF 12-MCS ranges from 0 [worst] to 100 [best]

**Table 6:** WOMAC-Index and associated patients’ characteristics and clinical conditions: multivariable generalised estimating equation models

WOMAC–Index	Total (***n*** = 132)			Rehabilitation program after discharge					
				Yes (***n*** = 40)			No (***n*** = 92)		
	β	95%CI	P	β	95%CI	P	β	95%CI	P
Intercept	31.45	20.71,42.18	< 0.001	32.70	11.44,53.95	0.003	30.19	19.50,40.87	< 0.001
Time (3-months after surgery)	−19.66	−31.77,-7.55	**0.002**	−10.48	−37.13,16.17	0.441	−20.65	−32.82,-8.49	**0.001**
**Age ≥ 65** years	−3.94	−9.71,1.84	0.181	−1.69	− 13.80,10.43	0.785	−4.46	−11.10,2.17	0.188
Time*Age ≥ 65 years	4.23	− 2.87,11.34	0.243	4.43	−8.65,17.51	0.507	3.46	−4.87,11.79	0.416
**Female sex**	10.18	4.48,15.89	**0.001**	6.12	−5.56,17.80	0.304	12.67	6.15,19.19	**< 0.001**
Time*Female sex	−4.74	−11.10,1.63	0.145	− 1.66	− 15.31,11.99	0.811	−7.73	− 14.59,-0.87	**0.027**
**Low education**	3.65	−1.65,8.95	0.177	12.28	1.41,23.14	**0.027**	0.12	−5.85,6.09	0.969
Time*Low education	3.88	−2.99,10.74	0.269	−2.92	−15.18,9.35	0.641	7.47	0.09,14.84	**0.047**
**BMI ≥ 30** kg/m^2^	4.58	−0.41,9.58	0.072	7.95	−2.66,18.56	0.142	5.70	0.13,11.27	**0.045**
Time*BMI ≥30 kg/m^2^	−5.97	−11.78,-0.16	**0.044**	−13.46	−25.23,-1.69	**0.025**	−6.44	−13.08,0.21	**0.058**
**Depressive symptoms**	11.47	5.03,17.92	**0.001**	11.90	−0.78,24.59	0.066	11.01	4.45,17.58	**0.001**
Time*Depressive symptoms	−2.69	−9.85,4.48	0.463	−3.57	−17.73,10.59	0.621	−3.66	−11.65,4.33	0.370
**Cognitive impairment**	−3.02	−10.51,4.47	0.429	−3.52	−21.32,14.27	0.698	−3.90	−12.73,4.93	0.387
Time*Cognitive impairment	−7.64	−15.42,0.13	**0.054**	−18.53	−42.57,5.50	0.131	−3.78	− 12.21,4.65	0.380
**Comorbidity ≥ 4 diseases**	−1.69	−7.15,3.76	0.543	−0.66	−12.25,10.94	0.912	−0.34	−6.43,5.76	0.914
Time*Comorbidity ≥4 diseases	−2.77	−9.56,4.01	0.423	− 9.64	−22.00,2.73	0.127	−2.69	−10.52,5.13	0.500
**Current smoker**	−2.40	−10.67,5.87	0.569	−22.79	−44.47,-1.11	**0.039**	1.58	−5.61,8.77	0.666
Time*Current smoker	−2.29	−11.62,7.04	0.630	25.41	9.20,41.61	**0.002**	−7.55	−16.24,1.14	0.089
**Current alcohol user**	−2.19	−8.02,3.64	0.462	−6.98	−18.46,4.50	0.233	−2.62	−8.41,3.16	0.374
Time*Current alcohol user	5.09	−2.28,12.47	0.176	6.69	−9.03,22.41	0.404	6.28	−1.18,13.75	0.099
**TKA surgery**	9.86	4.51,15.22	**< 0.001**	9.71	−2.02,21.44	0.105	11.11	5.60,16.63	**< 0.001**
Time*TKA surgery	−6.33	−12.82,0.17	**0.056**	−8.20	−21.54,5.15	0.229	−7.03	−14.11,0.04	**0.052**

Abbreviation: β, coefficient regression, *CI* confidence intervals, *WOMAC* Western Ontario and McMaster Universities Arthritis Index, *BMI* Body Mass Index, *TKA* total knee arthroplasty (reference UKA, unicompartmental knee arthroplasty)

WOMAC-Index is normalised to 100, ranges from 0 [best] to 100 [worst]

#### PCS of SF-12

The PCS (Table 4) worsened over time in the older patients, but it improved in the obese patients and in those with 4 and more comorbid diseases. The negative association between depressive symptoms and PCS did not change over time.

When the data of the patients who underwent rehabilitation after discharge were analysed, the PCS worsened over time in the older patients and current smokers; however, it improved in the patients with 4 or more diseases. It was unchanged over time in the patients who underwent TKA and with depressive symptoms.

#### MCS of SF-12

The MCS (Table 5) improved over time in the patients with low education, but it was unchanged in those with a high degree of comorbidity and depressive symptoms.

When the data of the patients who underwent rehabilitation after discharge were analysed, the MCS was found to improve over time in those with ≥4 diseases and in the current alcohol users; it was unchanged in the presence of depressive symptoms and in the low education (negative associations) and cognitively impaired patients (positive association).

#### WOMAC-Index

The WOMAC-Index scores (Table 6) improved in a marginally significant manner over time in the obese patients. They were unchanged in the presence of depressive symptoms in the patients who underwent a total knee replacement.

When the data of the patients who underwent rehabilitation after discharge were analysed, it was found that pain, stiffness and disability perception improved in the obese patients but worsened over time in the current smokers.

When the data of the patients who did not undergo rehabilitation after discharge were analysed, it was found that pain, stiffness and disability perception improved in the females, in the obese patients, and in those who underwent a TKA. They worsened instead over time in the patients with low education; they were unchanged in the presence of depressive symptoms.

## Discussion

This study aimed to examine the QOL of patients with knee OA, particularly in patients at high risk of functional decline and with relevant comorbidity burdens and high levels of polypharmacotherapy, at the time of and following knee replacement surgery. At baseline, the women had worse general health status, both physical and mental, and reported more pain and made more complaints about OA with respect to men [22].

The patients’ QOL was improved 3-months after surgery according to both the SF-12 and the WOMAC–Index, confirming that knee replacement *surgery* can determine a substantial improvement in QOL in patients with end-stage OA [1]. Analysis of the relationship between the baseline characteristics and the various endpoints revealed that higher BMI, higher comorbidity and TKA surgery were the variables associated with improvement. The fact that the patients affected by obesity and with ≥4 comorbid conditions showed considerable improvement in physical function and reported less pain is not surprising. Obese patients, who are frequently characterised by difficulty in walking and function, certainly benefit the most from surgery that permits them to increase their physical activity. The same can be said for comorbidities, as individuals affected by the typical physical impairments associated to cardiovascular and other frequent chronic conditions surely benefit from improved lower limb physical function.

Patients with knee OA are characterised by slow walking gait, fatigue and low levels of physical activity. As the main symptom of OA is joint pain, which is exacerbated by exercise and relieved by rest, it strongly affects the individual’s ability to perform the basic ADLs and often leads to frailty and disability [23, 24]. It is known that knee OA and frailty share common risk factors, such as obesity and comorbidities. The finding that patients with knee OA and these additional conditions benefit from knee replacement surgery is important because it demonstrates that knee replacement surgery can contribute to preventing frailty, a highly prevalent condition that leads to an increased risk of disability, falls, hospitalisation and death [25].

An analysis of study data uncovered that depressive symptoms at baseline was associated to higher self-reported pain, stiffness and disability, and this association remained unchanged at the 3-month post-surgery assessment. These findings underline the importance of depressive symptoms and their strong impact on the QOL of OA patients, independent from the type of surgical treatment or participation in a rehabilitation program. A randomised clinical trial evaluating 1801 depressed older patients with OA demonstrated that pharmacological treatment and/or psychotherapy had a significant beneficial effect not only on the depressive symptoms, but also on QOL, physical function and perception of OA-linked pain. Given the high co-prevalence of depressive symptoms in OA elderly patients presumably leading to worse QOL, the hypothesis that monitoring and treating depression could lessen the burden of OA in these patients seems more than reasonable [26].

In the management of knee OA, the efficiency of replacement surgery compared with alternative conservative strategies has been proven [27]. However, debate continues over what the most effective type of prosthesis is for the treatment of symptomatic primary medial compartment OA. Several advantages of UKA over TKA, including preservation of bone stock, faster recovery, lower overall cost, reduced morbidity, better functional outcome because of more normal knee kinematics, and subjective feeling of a more natural knee [28–35]. The main problem as far as UKA is concerned, is the higher revision rate, particularly in younger patients, with respect to TKA [36–39].

In our study, the type of implant was found to be a predictor of improvement at the 3-month post-surgery assessment. Specifically, TKA was significantly associated to an improvement in QOL. We believe that these results were probably due to a response shift bias, because patients who underwent TKA were experiencing more pain and disability *prior* to the surgery and thus perceived more improvement in function and pain with respect to their partial-surgery counterparts. OA radiological severity, postoperative complications, preoperative clinical parameters and comorbidities were defined common predictors for 5-year outcomes after knee replacement [27]. Hence, worse preoperative function and radiological severity of OA are generally associated with better postoperative improvement after TKA and greater patient satisfaction. Return to daily activity continues to be a key factor after knee arthroplasty. Patients often have expectation about being able to return to the activities they enjoyed prior to their limitations caused by knee OA.

Among the patients who did not give up the rehabilitation program after discharge, the current smokers reported worse physical function and pain symptoms. Patients are usually encouraged to carry out bending and straightening exercises as well as flexing and relaxing thigh muscles during rehabilitation programs after knee replacement surgery, which can last several months. They are also asked to walk progressively longer distances and to gradually include some resistance training.

The perception of worse physical function, pain and disability at three-months after surgery reported by the smokers might be due to the fact that they have greater ambulatory dysfunction associated in part to their higher CVD and comorbidity rates. Although it has been demonstrated that smokers benefit from rehabilitation programs as much as non-smokers, the benefits are nevertheless usually evident after at least a 6-month period. Despite the fact that exercise effectively seems to improve functional independence in both smoking and non-smoking patients, the former may report worse physical function and more pain over only a 3-month post-surgery period with respect to the latter due to smokers’ higher rates of vascular diseases [40].

Moreover, among the behavioural factors, smoking is the best-studied risk factor in TKA [41–43], responsible for a higher risk of infection, surgical complications and mortality. In particular, some studies [44, 45] suggest that smoking may be associated with increased risk of aseptic loosening due to delayed bone healing and bone regeneration, which leads to subsequent revision of the implant. The data reported by Kunutsor et al. [46] showed a generally increased risk of periprosthetic joint infection (PJI) after total joint arthroplasty in smokers compared to non-smokers. Smoking can cause endothelial dysfunction, inflammation, progression of atherothrombosis and impaired systemic immune response, which are known to contribute to poor wound healing and subsequently to infection. However, following total knee arthroplasty, there is no evidence for an association between smoking and the risk of revision surgery [47], and smoking status does not seem to impact hospitalisation length of stay [48].

Just as for smoking, high alcohol intake is known to lead to higher postoperative complications, including PJI. However, no statistically significant association of age or high alcohol intake with risk of PJI has been described after total joint arthroplasties [46], while a significantly lower risk of revisions among patients who were at least moderate drinkers has been reported [57].

Although the results of this study were presented a few years after patient evaluation, they are still relevant because currently, similar surgical indications are given for individuals with knee OA, and the same knee prosthetic implant and rehabilitation protocols are still used.

One potential source of bias in our study was the use of only self-reported measurements of physical function and not performance-based tests, which are less influenced by psychologic factors, cognitive impairments and educational level [49]. Another limitation is that the study was single-centre based, meaning that its results should be confirmed by other investigations. Finally, although many traditional predictors of physical function were examined, the change from baseline values may have been affected by unmeasured variables that were not included in our analysis.

One of the study’s strengths was that we calculated a sample size that guaranteed a sufficient statistical power as opposed to most studies in this field characterised by small sample size [14]. We were, moreover, in the position to assess most of the traditional behavioural, demographic, biological and physical risk factors for physical function, permitting us to identify subgroups, such as current smokers, for a less biased interpretation of the overall results.

## Conclusions

Our study shows that in patients with end-stage OA undergoing knee replacement surgery, 3-months after surgery QOL and physical function improved, according to both the SF-12 and the WOMAC–Index.

These findings demonstrate that surgery represents a valid approach to severe OA at any age, and that a comprehensive assessment, including patient-reported symptoms and outcomes, can help to identify risk and protective factors associated to physical function and QOL. Moreover, this study suggests that TKA should be considered the best surgical approach in case of severe knee OA pain, particularly in obese patients, irrespectively of age and OA stage.

## Data Availability

The datasets used and/or analysed during the current study are available from the corresponding author on reasonable request.

## Abbreviations

ADLs: Activities of Daily Living
BMI: Body Mass Index
CI: Confidence interval
GDS: Geriatric Depression Scale
GEE: Generalised estimating equation
IQR: Interquartile range
MCS: SF-12 Mental component
MMSE: Mini-Mental State Examination
OA: Osteoarthritis
PCS: SF-12 Physical component
PJI: Periprosthetic joint infection
QOL: Quality of life
QPro-Gin: Quality of Life in Knee Prostheses
ROM: Range of motion
SD: Standard deviation
SE: Standard error
SF-12: Short-Form General Health Survey
TKA: Total knee arthroplasty
UKA: Unicompartmental knee arthroplasty
WOMAC: Western Ontario and McMaster Universities Arthritis Index
